# CD8^low^ T cells expanded following acute *Trypanosoma cruzi* infection and benznidazole treatment are a relevant subset of IFN-γ producers

**DOI:** 10.1371/journal.pntd.0008969

**Published:** 2020-12-21

**Authors:** Alessandro Marins-Dos-Santos, Bianca Perdigão Olivieri, Rafaella Ferreira-Reis, Juliana de Meis, Andrea Alice Silva, Tania C. de Araújo-Jorge, Joseli Lannes-Vieira, Vinicius Cotta-de-Almeida

**Affiliations:** 1 Laboratory on Thymus Research, Oswaldo Cruz Institute, FIOCRUZ, Rio de Janeiro, Brazil; 2 Laboratory for Innovations on Therapy, Education and Bioproducts, Oswaldo Cruz Institute, FIOCRUZ, Rio de Janeiro, Brazil; 3 National Institute of Science and Technology on Neuroimmunomodulation (INCT-NIM), Rio de Janeiro, Brazil; 4 Department of Pathology, School of Medicine, Federal Fluminense University, Niterói, Brazil; 5 Laboratory of Biology of the Interactions, Oswaldo Cruz Institute, FIOCRUZ, Rio de Janeiro, Brazil; Universidade Federal de Minas Gerais, BRAZIL

## Abstract

CD8 T cells are regarded as pivotal players in both immunoprotection and immunopathology following *Trypanosoma cruzi* infection. Previously, we demonstrated the expansion of CD8^+^ T lymphocytes in the spleen of *T*. *cruzi*-infected mice under treatment with benznidazole (N-benzyl-2-nitroimidazole acetamide; Bz), a drug available for clinical therapy. This finding underlies the concept that the beneficial effects of Bz on controlling acute *T*. *cruzi* infection are related to a synergistic process between intrinsic trypanocidal effect and indirect triggering of the active immune response. In the present study, we particularly investigated the effect of Bz treatment on the CD8^+^ T cell subset following *T*. *cruzi* infection. Herein we demonstrated that, during acute *T*. *cruzi* infection, Bz treatment reduces and abbreviates the parasitemia, but maintains elevated expansion of CD8^+^ T cells. Within this subset, a remarkable group of CD8^low^ cells was found in both Bz-treated and non-treated infected mice. In Bz-treated mice, early pathogen control paralleled the lower frequency of recently activated CD8^low^ cells, as ascertained by CD69 expression. However, the CD8^low^ subset sustains significant levels of CD44^high^CD62L^low^ and CD62L^low^T-bet^high^ effector memory T cells, in both Bz-treated and non-treated infected mice. These CD8^low^ cells also comprise the main group of spontaneous interferon (IFN)-γ-producing CD8^+^ T cells. Interestingly, following *in vitro* anti-CD3/CD28 stimulation, CD8^+^ T cells from Bz-treated *T*. *cruzi*-infected mice exhibited higher frequency of IFN-γ^+^ cells, which bear mostly a CD8^low^ phenotype. Altogether, our results point to the marked presence of CD8^low^ T cells that arise during acute *T*. *cruzi* infection, with Bz treatment promoting their significant expansion along with a potential effector program for high IFN-γ production.

## Author summary

Chagas disease is a neglected illness caused by the protozoan *Trypanosoma cruzi*, which affects 6 to 7 million people worldwide. The current treatment for acutely-infected patients is mostly limited to the benznidazole (Bz) drug, reaching up to 80% of cure. It has been proposed that Bz therapy efficacy involves both trypanocidal and immunonodulatory effects. In this sense, we previously suggested that CD8^+^ T cells, highly expanded after Bz treatment of acute *T*. *cruzi*-infected mice, might play a particular role in parasite control. Here, by further investigating those expanded CD8^+^ T cells, we observed that they bear a clear-cut effector phenotype and that a significant part of them stand out as a subpopulation bearing low levels of CD8 on their surface. Interestingly, besides the evident parasite control, Bz-treated mice sustain a group of effector CD8^low^ cells with spontaneous IFN-γ production. Moreover, *in vitro*-stimulated CD8^+^ T cells, sorted from infected and Bz-treated mice, present a relevant group of IFN-γ^+^ cells with a CD8^low^ phenotype. Altogether, our data indicate the particular subset of CD8^low^ cells as potentially contributing for a sustained protective immunity in *T*. *cruzi-*infected animals under Bz therapy.

## Introduction

*Trypanosoma cruzi* is the etiologic agent of Chagas disease, a neglected illness considered a global health problem which affects around 6–8 million people worldwide [1,2]. Noteworthy, countries with vectorial transmission of Chagas disease are also reporting outbreaks of oral infection by ingestion of contaminated food [3,4]. Once infected, the host immune system generates a potent inflammatory response that includes cytokines such as IFN-γ and tumor necrosis factor (TNF), which act synergistically and promote parasitic load control during the acute phase of infection [5–7]. Although the immune response reduces the parasite burden, the infection is not eliminated [8]. In fact, parasite persistence [9] is likely to contribute to the effects of long-term *T*. *cruzi* infection, which might induce gradual progression towards a severe symptomatology observed in a high proportion of infected individuals [10]. Remarkably, CD8^+^ T cells have been regarded as critical players for both immunoprotection and immunopathological disturbances observed in experimental and clinical studies [11–14].

Interestingly, experimental studies have demonstrated that the beneficial activity of the trypanocidal medicament benznidazole (Bz) on parasitological cure seems to be dependent on IFN-γ and partially dependent on TNF, IL-12 and nitric oxide [15]. Additionally, our previous investigation indicated that Bz treatment might enhance cellular immunity through expansion of activated CD8^+^ T cells, reversal of thymus atrophy and induction of resistance to reinfection [16,17]. Herein, we sought to explore the importance of the highly expanded CD8 subset by further characterizing these cells following acute *T*. *cruzi* infection and Bz treatment.

## Material and methods

### Ethics statement

Female C57BL/6 mice (aged 6 to 8 weeks) were maintained under specific pathogen free (SPF) conditions at Oswaldo Cruz Institute animal facilities. All procedures of animal handling carried out in this work were performed according to the rules of the Animal Ethics Committee of the Oswaldo Cruz Foundation (CEUA Fiocruz L-29/14).

### Infection and benznidazole treatment

*T*. *cruzi* infection was carried out by intraperitoneal inoculation with 10^4^ bloodstream trypomastigotes of Y strain in 200 μl of phosphate-buffered saline (PBS). These parasites were collected from blood of infected albino Swiss mice at peak of parasitemia by differential centrifugation, as described previously [16]. Mice were treated *ad libitum* by the addition of 0.25 mg of Bz per mL to the drinking water from 7 to 14 days post-infection (dpi), for a calculated daily dosage of 100 mg/Kg of body weight [16]. Fresh Bz solution was added every day in drinking water. Decrease of circulating parasites in bloodstream was correlated with treatment efficacy. The following groups were employed in this work: non-infected and non-treated (NI), infected non-treated (I) and infected and benznidazole-treated (IBz).

### Parasitological parameters

Parasitemia was monitored by taking 5 μL of fresh blood from the mouse tail followed by gentle compression between glass slide and coverslip (18 x 18 mm). The number of parasites per mL was determined by scoring 50 fields, and that number was multiplied by a conversion factor which takes into account the number of microscopic fields in the area under specific magnification.

### Reagents and antibodies

N-Benzyl-2-nitroimidazole acetamide, benznidazole, (Lafepe, Pernambuco, Brazil) was used as a trypanocidal drug in the experimental therapy schedules. The following anti-mouse monoclonal antibodies (mAbs) were used for phenotyping lymphocyte by flow cytometer analysis: anti-CD45R PE-CF594 clone RA3-6B2, anti-CD3 PE clone 17A2, anti-CD4 PerCp clone RM4-5, anti-CD4 PE-Cy7 clone GK 1.5, anti-CD8 APC-Cy7 clone 53–6.7, anti-CD62L FITC clone Mel 14, anti-T-bet PE-Cy7 4B10 (all from eBioscience, San Diego, CA, USA), and anti-CD8 PE clone 53–6.7, anti-CD44 PE clone IM7, anti-CD69 PE clone HI.2F3, anti-CD8 APC clone 53–6.7, anti-IFN-γ APC clone XMG1.2 (all from BD Pharmingen, San Diego, CA, USA). PE-Cy7-conjugated mouse IgG1 kappa (eBioscience) and APC-conjugated rat IgG1 kappa (BD Pharmingen) were used, respectively, as isotype controls for T-bet and IFN-γ stainings. Purified antibody rat anti-mouse CD16/CD32 clone 2.4G2 (BD Pharmingen) was employed for Fc receptor blocking. Intracellular labeling was performed by employing the BD Cytofix/Cytoperm Plus Kit with BD Golgiplug protein transport inhibitor (BD Bioscience, San Jose, CA, USA). For splenocyte culture, RPMI 1640 medium was used supplemented with fetal bovine serum (FBS) (Cultilab, São Paulo, Brazil), HEPES, L-glutamine, β-mercaptoethanol, non-essential amino acids, sodium pyruvate, Phorbol 12-myristate 13-acetate and ionomycin (all from Sigma-Aldrich, St. Louis, MO, USA); purified anti-CD3 and anti-CD28 (both from BD Pharmingen) were utilized for activation in cell culture.

### Lymphocyte phenotyping analysis

Cell suspension containing 1 x 10^6^ splenocytes from each animal were submitted to erythrocyte lysis and filtered through a 70-μm cell strainer (BD Falcon), followed by washing and staining with Fc receptor blocking. After 10 minutes, cells were centrifuged and resuspended for a final incubation with appropriate dilutions of the mAbs for surface marker phenotyping. Intracellular staining was performed according to the manufacturer's instructions. After all immunostainings, cells were washed and immediately analyzed by flow cytometry. Data acquisition to four-color to seven-color analysis was performed on a FACSCanto (BD Bioscience) or CyAn (Dako cytomation) flow cytometers. A total of 20,000 events were collected in lymphocyte-enriched region based in morphological parameters gating (CD3^+^ backgate was used for better region visualization), for lymphocyte subset characterization. The flow cytometry acquisition of 100,000 events was performed for improve activation and cytokine evaluations. The positive regions were defined, after gating in singlets, by the limit of autofluorescence found in isotype control or unlabeled cells, and the compensation test was done using Fluorescence Minus One (FMO) method. Further data analysis was carried out with Summit or Kaluza (Beckman Coulter) flow cytometry software. Gating strategy for the analysis of the distinct lymphocyte subsets is provided (S1 Fig).

#### Cell Sorting and functional characterization of CD8^+^ cells

CD8^+^ cells were sorted by positive selection via flow cytometry (MoFlo Legacy cell sorter, Beckman Coulter). Splenocytes were immunostained with anti-CD4 PE-Cy7 and anti-CD8 APC, respectively, for exclusion and selection. The pool of CD8^+^ cells purified from five mice of each experimental group were maintained in culture in 96-well U-bottom plates at a concentration of 2 x 10^5^ cells per well. Cell culture medium was RPMI 1640 supplemented with 10% FBS, 10 mM HEPES, 1% L-glutamine, β-mercaptoethanol, 1% non-essential amino acids and 1 mM sodium pyruvate. Cells were maintained in culture for 36 h, at 37°C and 5% CO_2_, with plate-coated purified anti-CD3 (10 μg/mL; BD Pharmingen) and with soluble purified anti-CD28 (1 μg/mL; BD Pharmingen). Assessment of IFN-γ production by these purified and *in vitro-*activated CD8^+^ T cells was performed by flow cytometer analysis, as previously described. Gating strategy for sorting and further analysis of the sorted CD8^+^ cells is provided (S2 Fig).

### Statistical analysis

All statistical analysis was done in GraphPad Prism 6 (GraphPad Software Inc.). Data were subjected to the Shapiro normality test to determine whether they were sampled from a Gaussian distribution. Kruskal-Wallis with Dunn’s multiple comparison test or ANOVA with Tukey’s multiple comparison test were applied for samples that, respectively, deviated or assumed a Gaussian distribution after. Outlier was calculated by the Grubbs' test. When P < 0.05, values were considered statistically significant.

## Results

### Benznidazole treatment of infected mice controls parasite burden, but maintains elevated splenocyte number

Analysis of circulating parasites showed that infected and non-treated mice (I group) manifest rapid parasite expansion between 6–8 days post-infection (dpi), with parasitemia peak at 8 dpi. In contrast, Bz treatment initiated at 7 dpi shortened the infection course (IBz group), with reduction in parasite number (S3A Fig). We also searched for alterations in spleen cellularity, and our results show significant increase in splenocyte number at 14 dpi in both infected groups, as compared to the control non-infected and non-treated mice (NI group) (S3B Fig). However, analysis of the spleen cellularity by normalizing the number of splenocytes per spleen mass revealed no significant difference among the groups and some variations between biological replicates (S3C Fig). As previously defined, Bz treatment of non-infected mice did not affect the lymphocyte distribution as compared to non-treated and non-infected mice [16,17].

### CD8^low^ cells are highly expanded during the acute infection and maintained with benznidazole treatment

**Fig 1:**
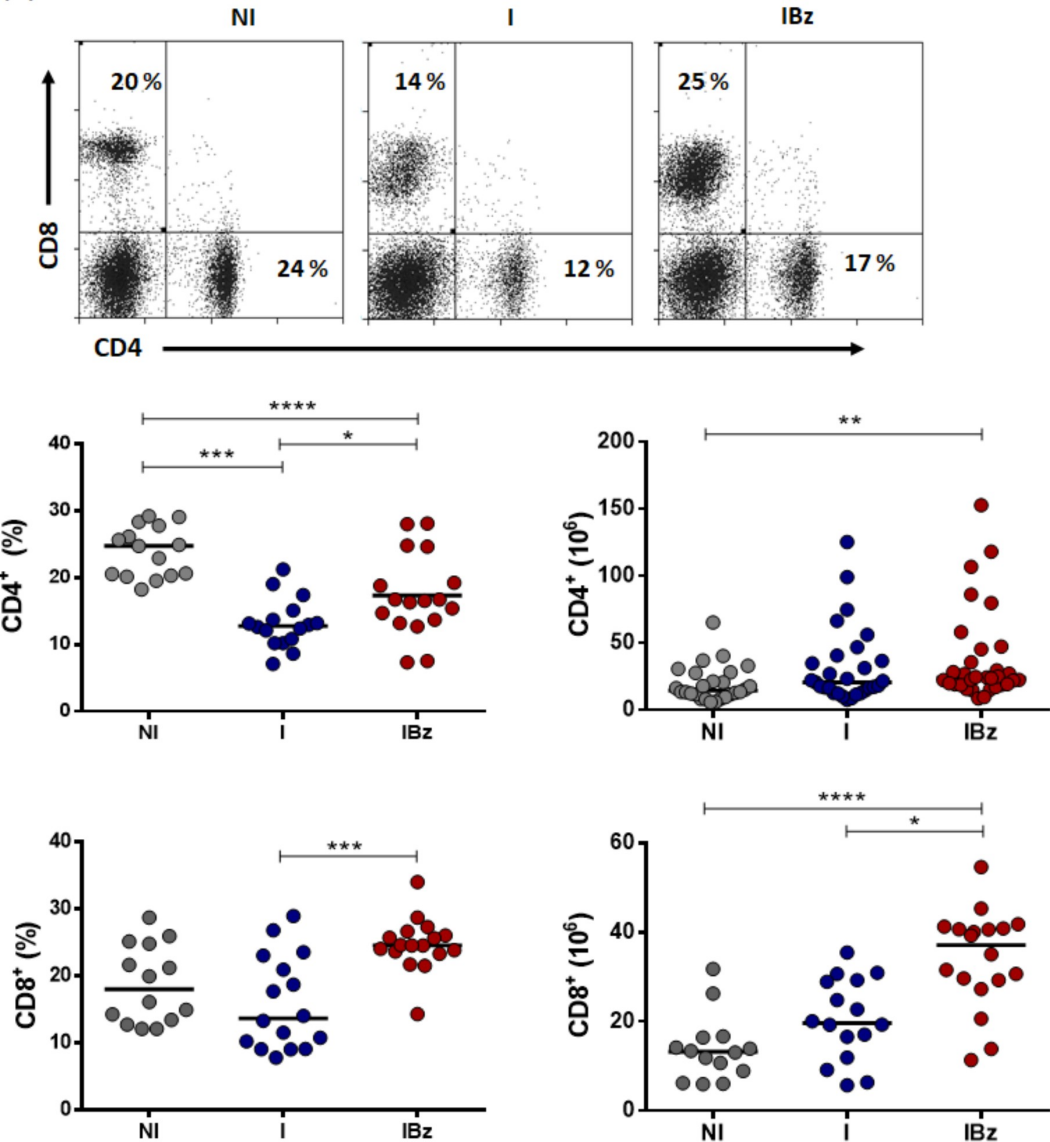
CD8 cells expanded after infection and benznidazole treatment. Distribution of CD4 and CD8 subpopulations are shown by representative dotplots and individual frequency data from four experiments, each with n = 3–4 mice in non-infected and non-treated (NI) group, n = 3–5 mice in infected non-treated (I) and infected and benznidazole-treated (IBz) groups; absolute numbers are from seven experiments, each with n = 3–5 mice in each group.

**Fig 2:**
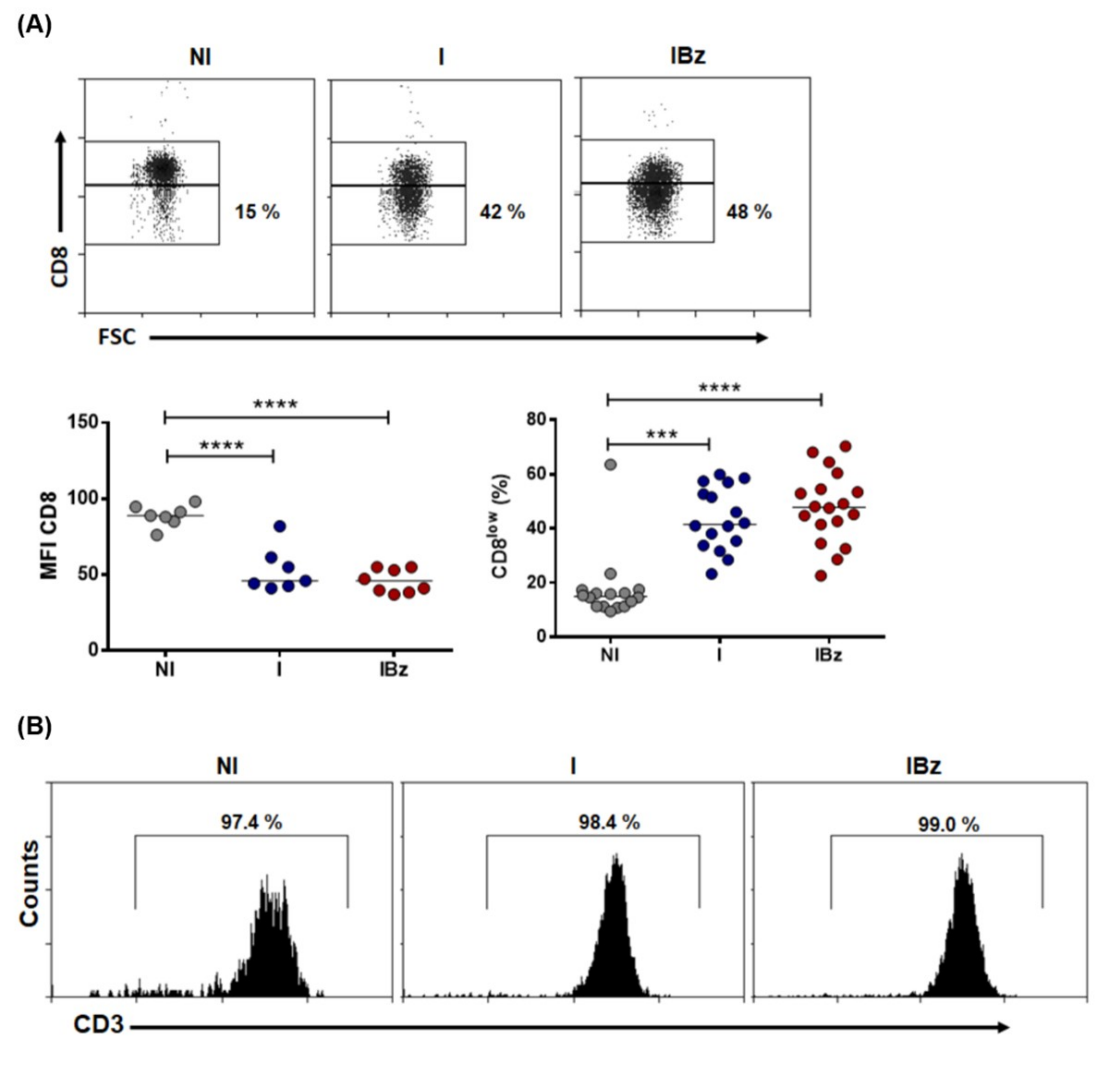
Expanded CD8 cells comprise a remarkable CD8^low^ subset. (A) Frequency of CD8^low^ cells were analyzed by gating total CD8^+^ cells (representative dotplots) and represent data collected from 4 experiments with 3–5 mice in NI, I and IBz groups. The CD8 Median Fluorescence Intensity (MFI) data are representative of two experiments with, respectively, n = 3 and 4 mice in NI and I groups, and n = 3 and 5 mice in IBz group. Absolute numbers of CD8^low^ were performed using data collected from 4 experiments with 3–4 mice in NI and I, and n = 3–5 mice in IBz per experiment. (B) Frequency of CD3^+^ cells were analyzed by gating total CD8^low^ cells. Data are from one experiment with n = 4 mice analyzed in NI and I groups, and n = 5 mice in IBz. The analysis were performed on day 14 post-infection; horizontal bars represent the average for each group; *p < 0.05, ** P <0.01, ***p < 0.001, **** P <0.0001 unpaired Kruskal-Wallis (Dunn's post-test).

Studies on experimental models and patients of Chagas disease suggested an interplay between trypanocidal effect of Bz and T-cell response against *T*. *cruzi* infection [15]. Herein, we search to characterize the expanded CD8^+^ T cells during the acute *T*. *cruzi* infection following Bz treatment. Analysis of lymphocyte subsets revealed that Bz-treated *T*. *cruzi*-infected mice presented a significant increase in the relative number of T cells paralleled to a decrease in the relative number of B cells (S3D Fig). However absolute numbers of B cells were increased in this IBz group (S3D Fig). Further analysis of CD4 and CD8 T cell subsets demonstrated a significant increase only in CD8^+^ cells within IBz group (Fig 1), as expected from our previously reported data [16]. Strikingly, our cytofluorometric analysis revealed a significant frequency of cells expressing low surface CD8 levels (phenotypically defined herein as CD8^low^) within the CD8^+^ T cell subset from both infected groups (I and IBz), when compared to the non-infected N group (Fig 2A). Our data also showed that these CD8^low^ splenocytes are highly expanded in the IBz group (Fig 2A), demonstrating that Bz treatment maintains the expansion of these cells, despite the early control of parasitemia. Noteworthy, by further gating this CD8^low^ subset, we observed that 98% of the cells are CD3^+^ (Fig 2B).

### Frequency of recently-activated CD8^low^ cells is reduced in benznidazole-treated mice, while an effector profile is maintained

**Fig 3:**
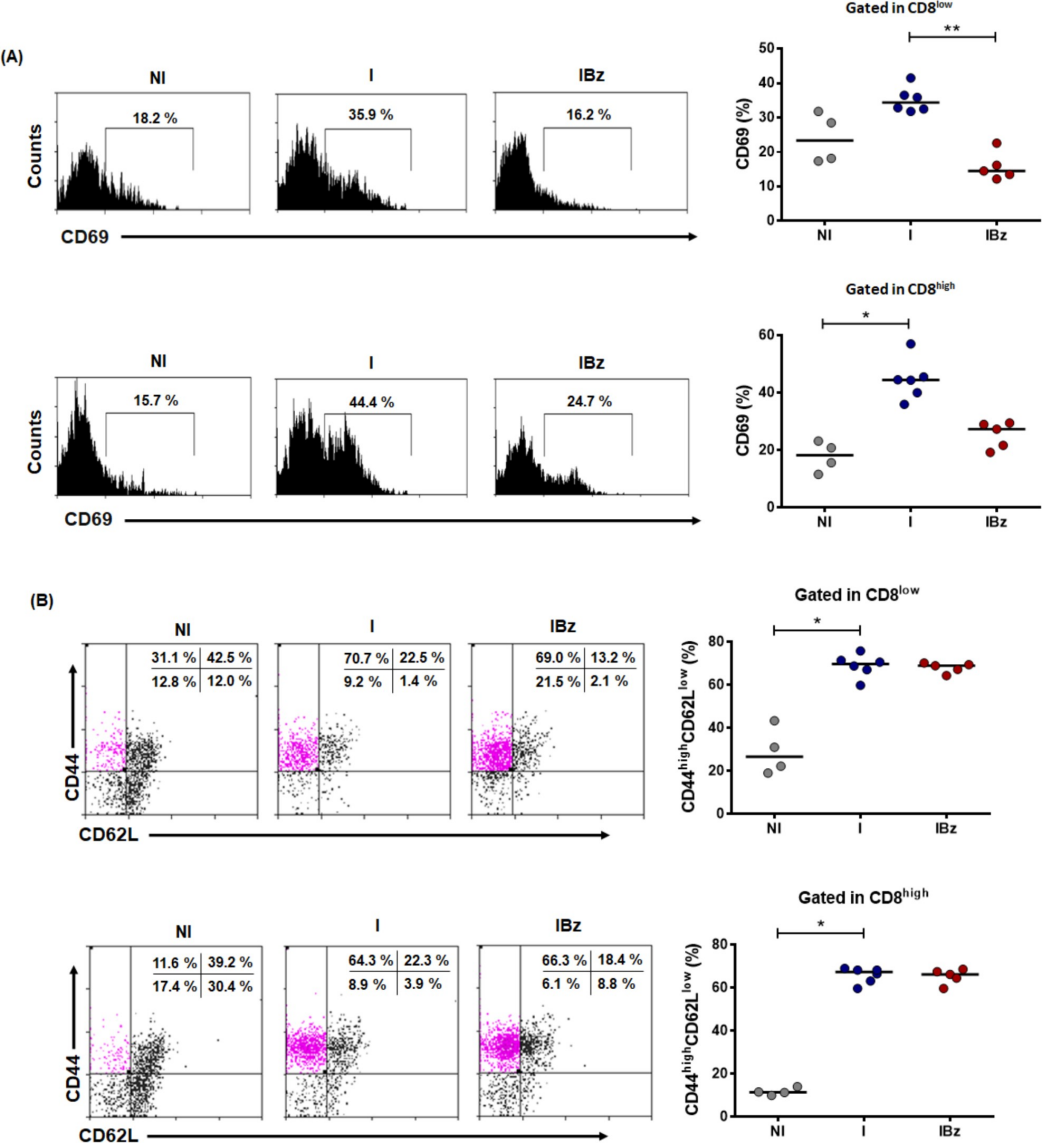
CD8^low^ subset of benznidazole-treated mice bear lower frequency of recently-activated CD69^+^ cells and increased frequency of effector CD44^+^CD62L^low^ cells. (A) Frequency of CD69^+^ cells within CD8^low^ and CD8^high^ subsets were analyzed from the groups non-infected and non-treated (NI) with n = 4, infected non-treated (I) with n = 6 and infected and benznidazole-treated (IBz) with n = 5. (B) Frequency of CD44^+^CD62L^low^ within CD8^low^ subset was analyzed from NI, I and IBz groups. (C) Frequency of CD44^+^CD62L^low^ within CD8^high^ subset was analyzed from NI with n = 4, I with n = 6 and IBz with n = 5. Respective representative histograms (CD69 fluorescence intensity) and dotplots (CD62L *versus* CD44) are shown. Data are from one experiment analyzed on day 14 post-infection; the horizontal bars represent the average for each group, *p < 0.05, ** P <0.01, unpaired Kruskal-Wallis (Dunn's post-test).

The striking finding of highly expanded CD8^low^ T cells prompted us to further characterize this subset. As determined by the CD69 expression, the I group presented an increased frequency of recently activated CD8^low^ cells, a finding not observed in the same subset from IBz group (Fig 3A). In addition, I group also showed increased numbers of CD8^high^CD69^+^ cells, as compared to the Bz-treated and infected animals. Importantly, analyses of CD44 and CD62L markers demonstrated a significant increase in the frequency of effector/memory CD44^high^CD62L^low^ cells within CD8^low^ and CD8^high^ subsets from both the untreated infected group and the Bz-treated infected group (Fig 3B and 3C). Similar finding was observed by analyzing the CD4 subset (S4 Fig).

**Fig 4:**
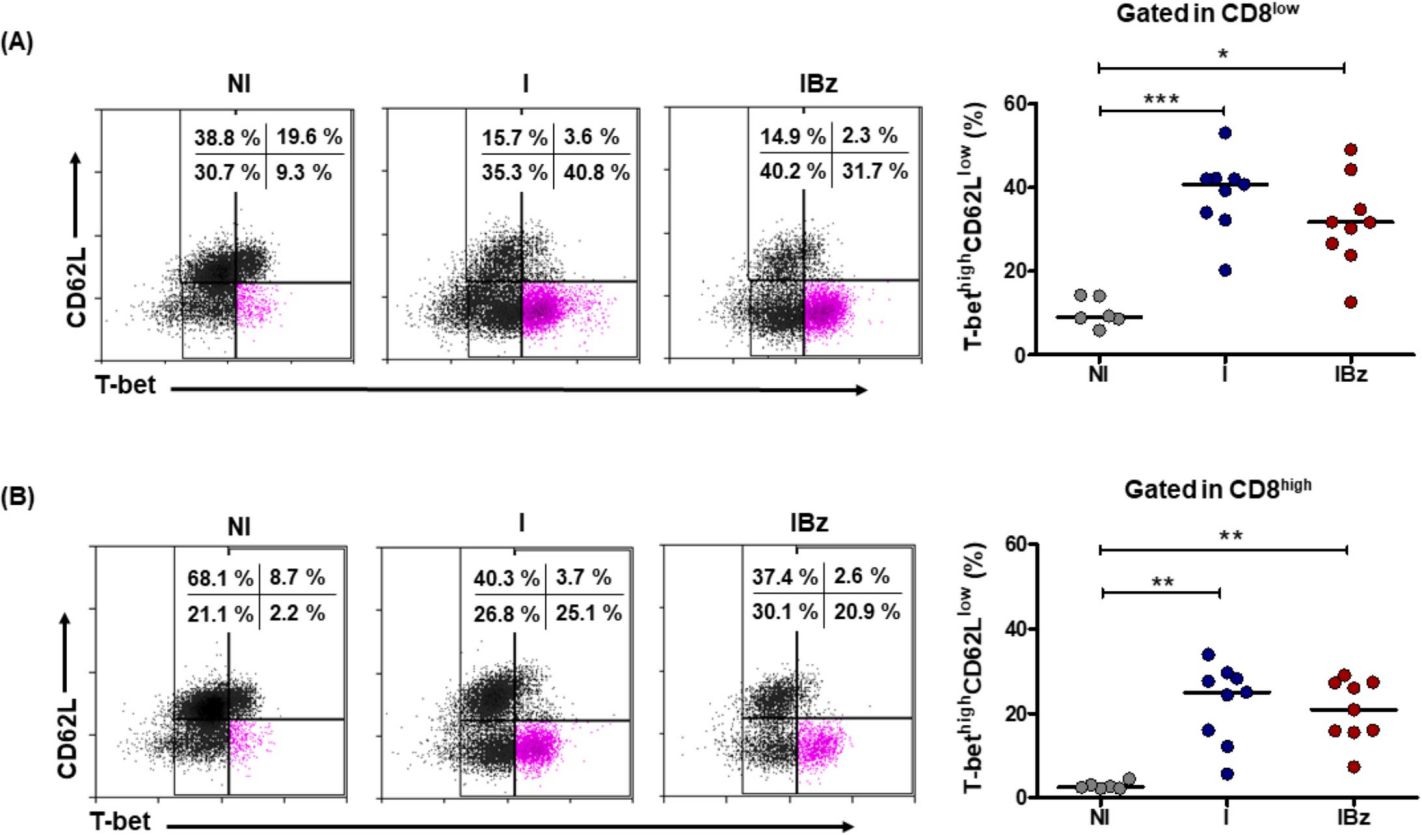
Benznidazole-treated mice display elevated frequency of CD8^low^CD62L^low^T-bet^high^ effector memory cells. (A) Frequency of CD62L^low^T-bet^high^ cells within CD8^low^ subset was analyzed from non-infected and non-treated (NI), infected non-treated (I) and infected and benznidazole-treated (IBz). (B) Frequency of CD62L^low^T-bet^high^ cells within CD8^high^ subset were analyzed from NI, I and IBz groups. Respective representative dotplots (T-bet *versus* CD62L) are shown. Data are from two experiments with n = 6 in NI group, and with n = 4 and 5 mice per experiment in I and IBz. Data were analyzed on day 14 post-infection; horizontal bars represent the average for each group; *p < 0.05, ** P <0.01, ***p < 0.001 unpaired Kruskal-Wallis (Dunn's post-test).

The understanding that CD8^low^ cells comprise a significant subgroup of effector cells was reinforced by assessing the intracellular expression of T-bet within the CD62L^low^ subpopulation. In fact, both infected groups showed more than two-fold increase in the frequency of CD8^low^CD62L^low^T-bet^high^ cells, as compared to N group (Fig 4A). Similar profile of activation was found within CD8^high^ cells of I group and IBz group (Fig 4B). Additionally, these analyses point out that central memory subsets CD62L^high^CD44^high^ and CD62L^high^T-bet^high^, within CD8^low^ and CD8^high^ T cells, are decreased in untreated *T*. *cruzi*-infected mice and such a decrease was not reverted after Bz treatment.

### CD8^low^ T lymphocytes from benznidazole-treated mice bear a relevant group of potential IFN-γ-producing cells

**Fig 5:**
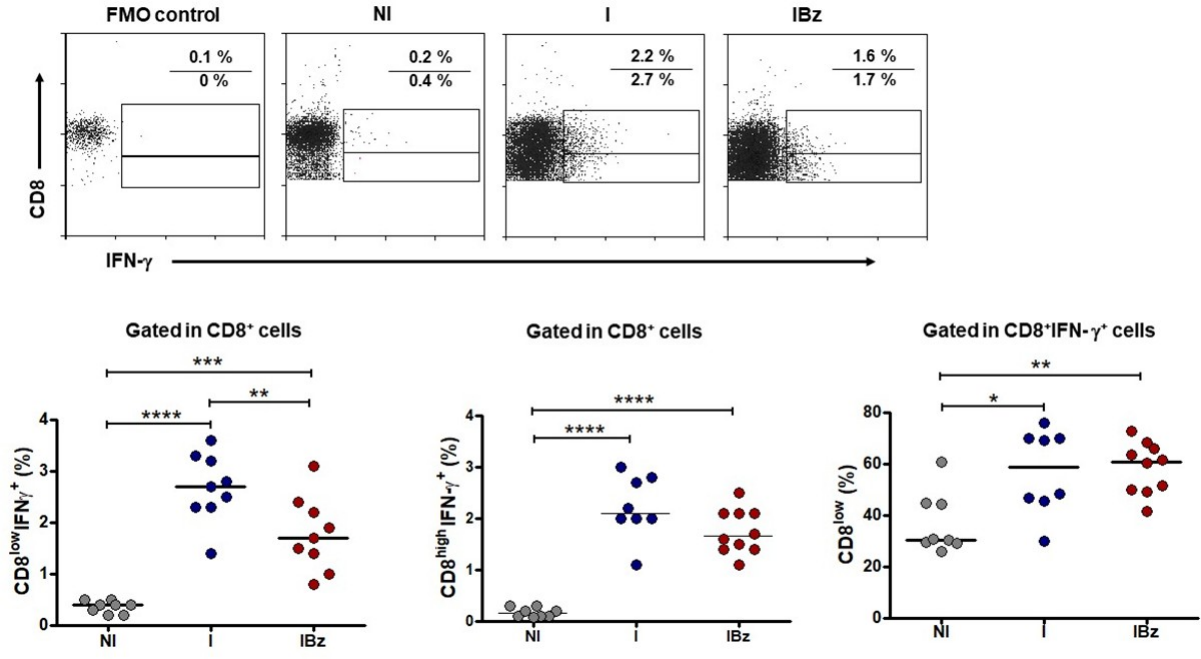
CD8^low^ cells are the main IFN-γ^+^ producers after infection and benznidazole treatment. CD8 cells from the of non-infected and non-treated (NI), infected non-treated (I) and infected and benznidazole-treated (IBz) groups were analyzed for the intracellular expression of IFN-γ. Representative dotplots of IFN-γ *versus* CD8 labeled cells gated in total CD8^+^ cells are shown. Rectangular gates define CD8^high^ and CD8^low^ subgroups, and the numbers in each dotplot represent the percentage of CD8^high^IFN-γ^+^ (top) and CD8^low^IFN-γ^+^ (bottom) cells. Graphs show individual frequency of CD8^low^IFN-γ^+^ cells (left) and CD8^high^IFN-γ^+^ cells (center) gated in CD8^+^ subset, as well as the frequency of CD8^low^ cells gated within total CD8^+^IFNγ^+^ cells (right). Data are from two experiments with n = 4 in N group, and with n = 4 and 5 mice per experiment in I and IBz. Data were analyzed at 14 dpi; horizontal bars represent the average for each group; *p < 0.05, ** P <0.01, ***p < 0.001, **** P <0.0001; unpaired Kruskal-Wallis (Dunn's post-test).

As CD62L^low^T-bet^high^ phenotype is pointed as prominently linked to IFN-γ production in T cells [18], we further assessed the IFN-γ production by CD8^low^ cells. In fact, these cells were found to be the main group of spontaneous IFN-γ producers within the whole CD8^+^ subset in both I and IBz group (Fig 5). Our results also showed that IBz group displayed significantly lower frequency of *ex-vivo* CD8^low^IFN-γ^+^ cells, when compared with the I group. Such a finding was not observed in the analysis of CD8^high^IFN-γ^+^ cells (Fig 5).

**Fig 6:**
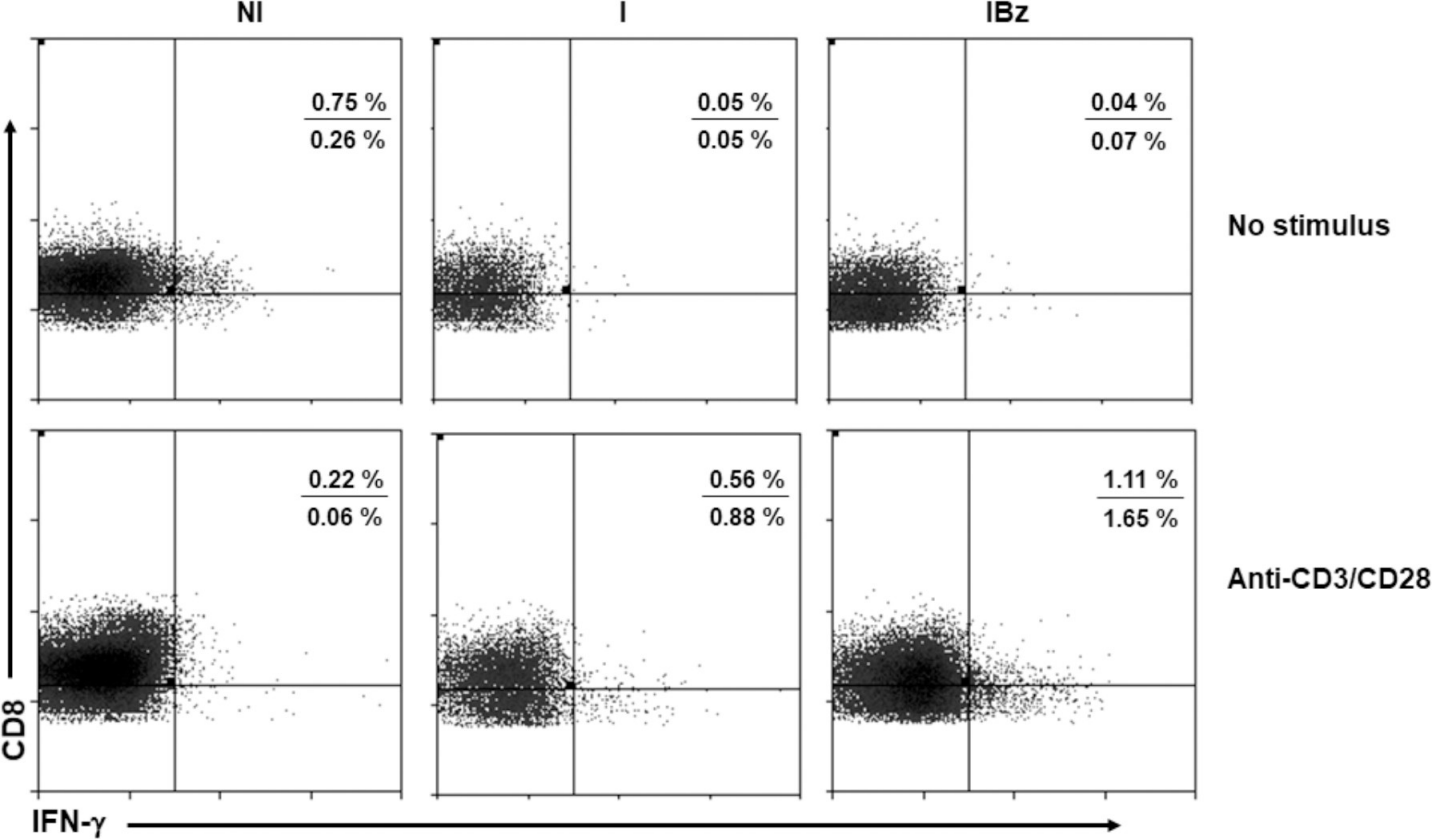
Benznidazole-treated mice have an increased frequency of IFN-γ-producing cells following *in vitro* stimulation. FACS-sorted CD8^+^ cells, from the groups of non-infected and non-treated (NI), infected non-treated (I) and infected and benznidazole-treated (IBz), mice were analyzed for the intracellular expression of IFN-γ following a 36-hour-culture without stimulus or stimulated with anti-CD3 and anti-CD28. Dotplots representing IFN-γ *versus* CD8 immunolabeling are shown. Numbers in each dotplot represent the respective percentages of CD8^high^IFN-γ^+^ (top) and CD8^low^IFN-γ^+^ (bottom) cells gated on total CD8^+^ cells. Data were analyzed on CD8^+^ cells sorted at 14 dpi and represent one experiment from a pool of 5 mice for each experimental group.

Moreover, by assessing the potential of CD8^+^ T cells to produce IFN-γ following anti-CD3 plus anti-CD28 *in vitro* stimulation, sorted CD8^+^ subset from both I and IBz groups showed increased frequency of IFN-γ-producing cells, as compared to control N group (Fig 6). However, the CD8^low^ subpopulation from Bz-treated *T*. *cruzi*-infected mice showed a more pronounced frequency of IFN-γ-producing cells, as compared to untreated *T*. *cruzi*-infected animals (Fig 6).

## Discussion

During the acute *T*. *cruzi* infection, CD8^+^ T cells are pivotal to establish the infection control by distinct mechanisms, particularly through the relevant production of IFN-γ [19–21]. Moreover, CD8^+^ T lymphocytes are a major group of immune cells within cardiac inflammatory sites in patients with chronic Chagas disease cardiomyopathy [22] as well as in chronically-infected mice [23]. In accordance, a cross-sectional analysis during chronic *T*. *cruzi* infection showed that the majority of patients bearing a less severe heart disease displayed increased frequency of circulating CD8^+^IFN-γ^+^ cells [12,13]. In fact, the number of IFN-γ-producing cells correlates with the lack of *T*. *cruzi* antigen detection within cardiac inflammatory lesions observed in chronic Chagas disease patients [24].

Nevertheless, fewer reports are available on the immunological effects of trypanocidal treatment in Chagas disease patients. This approach should always be considered, as the immune system seems to be a player for the mechanisms of Bz action [15,25]. Additionally, understanding the immunomodulatory effects of trypanocidal drugs might support the rational development of new anti-*T*. *cruzi* therapeutical approaches.

Previously, we showed that Bz treatment not only abbreviates the infection course by establishing parasite control, but it also elicits a preferential expansion of CD8^+^ T cells, as observed by an important increase of both relative and absolute numbers in the spleen of infected and Bz-treated mice [16]. Such a response might represent a protective systemic response against the infection, as the spleen harbors a diversity of clonotypic T cells able to respond and expand in an antigen-specific manner, protecting the host against pathogen dissemination [26].

Here, we demonstrated that a significant proportion of expanded CD8^+^ T cells bears a clear CD8^low^ phenotype following acute *T*. *cruzi* infection. Interestingly, Bz treatment of infected mice maintained a similar increased frequency of these particular subset of CD8^low^ cells, despite the controlled parasitemia and the lower levels of splenic recently activated CD8^+^ T cells. In fact, some studies have shown that a proportion of CD8^+^ T cells displays low surface expression of the CD8 molecule after viral infections [27–29]. During the early phase of human Hantaan virus (HTNV) infection, CD8^low^ cells expressed high levels of cytolytic effector molecules and were associated with high activation status, exhibiting an effector phenotype as CCR7^+/-^CD45RA^-^CD127^high^CD27^int^CD28^low^CD62L^low^ [30]. This study also revealed these cells as the main producers of IFN-γ and TNF among CD8^+^ T cells, when stimulated with specific HTNV peptides. Human CD8^low^ T cell subset seems to be in a more activated state, with increased proliferative response and both IFN-γ secretion and cytotoxic activity after stimulation [31]. In experimental *T*. *cruzi* infection, it was previously shown the presence of CD8^low^ cells following murine acute infection [32]. Moreover, CD8^low^ T cells seem to comprise a long-lasting subset found in chronically-infected mice [33]. Interestingly, the latter report points this subset as displaying low spontaneous cytokine secretion, but upon *in vitro* stimulation with *T*. *cruzi*-infected macrophages it is able to respond by producing IFN-γ [33].

As T-bet regulates the production of IFN-γ in T cells, with a subsequent role in mediating resistance to intracellular pathogen infections [18,34], we also investigated the *ex vivo* and *in vitro* production of IFN-γ by CD8 T cells. In fact, this effector cytokine is regarded as central for the balance between the generation of immune responses sufficient to control the *T*. *cruzi* infection and the regulation of this response to prevent extensive destruction of host tissues [11,14,35]. CD8^+^ T cell producing IFN-γ seems to be a central mediator of parasite control and protective immunity, while the cytolytic activity of CD8^+^ T cells has been reported as not required for the immunoprotective responses [36,37]. In this sense, reconstitution of *Cd8*^-/-^ mice with perforin-deficient CD8^+^ cells results in lower *T*. *cruzi*-elicited heart injury, whereas reconstitution with IFN-γ-deficient CD8^+^ cells shows aggravation of the cardiac lesions [11]. In addition, reduced production of IFN-γ by CD8^+^ T cells is also associated with increased severity of Chagas disease in humans [19].

Interestingly, our data revealed that CD8^low^ cells are the major spontaneous producer of IFN-γ following *T*. *cruzi* infection, including in the Bz-treated mice. In fact, the CD8^low^ cells from IBz group also showed a relevant IFN-γ production upon *in vitro* stimulation. As we did not analyzed distinct timepoints after *in vitro* stimulation, a complete understanding of this prompt IFN-γ production by the CD8^low^ cells from Bz-treated animals deserves further investigation. Another caveat for this finding is that, despite we showed the CD8^low^ subset as predominantly CD3^+^ cells, we cannot rule out the presence of CD8^low^ NK cells in the assays of IFN-γ production due to the absence of CD3 staining.

Nonetheless, these findings on spontaneous and polyclonally-stimulated IFN-γ production should be analyzed together with our data showing that CD8^low^ T cells in both untreated *T*. *cruzi*-infected and Bz-treated *T*. *cruzi*-infected mice might comprise a majority of cells bearing an effector memory phenotype. This conclusion can be supported by the findings of increased frequency of CD62L^low^CD44^+^ and CD62L^low^T-bet^high^ cells in these groups. Noteworthy, both groups also have similar numbers of cells bearing central memory phenotype, CD62L^high^CD44^+^ and CD62L^high^T-bet^high^. However, the lower CD69 expression following Bz therapy points to the notion that the CD8^low^ subset might represent resting effector memory T cells. Interestingly, these cells, likely originated after lowering of parasitemia and cessation of parasite-induced activation, bears a high capacity to promptly produce IFN-γ upon stimulation.

Ultimately, it is important to point out that down-regulation of CD8 expression in response to infections has been suggested as an inflammatory-derived regulation that limit potential tissue damage mediated by CD8^+^ T cells [38]. Therefore, in order to define whether Bz treatment might modulate the function of CD8^+^ T cells during acute *T*. *cruzi* infection, with a potential role in controlling both parasite infection and the cardiac immunopathology, it is necessary to assess specificity as well as polyfunctionality features of the CD8^low^ subset.

Moreover, as we previously reported that Bz-treated mice displayed resistance to reinfection [16], the present findings suggest that Bz treatment during the acute phase of *T*. *cruzi* infection might induce a subset of highly expanded CD8^low^ effector T cells with a high protective capacity. Likely, CD8^low^ T cells along with CD4 T cells as well as B lymphocytes might be critical subsets for an effective parasite control and increased survival of Bz-treated mice. Further functional studies to approach this hypothesis are deserved.

## Supporting information

S1 Fig Gating strategy for the analysis of the distinct lymphocyte subsets. (TIF)

S2 Fig Gating strategy for sorting and further analysis of the sorted CD8+ cells. (TIF)

S3 Fig Benznidazole treatment maintains elevated spleen cell number after controlling parasitemia. (A) Parasitemia levels were followed in mice from the infected non-treated (I) and infected and benznidazole-treated (IBz) experimental groups. (B) Absolute splenocyte number at 14 dpi was analyzed from groups of non-infected and non-treated (NI), I and IBz mice. (C) Spleen cellularity is shown as the ratio of splenocyte number (in millions) per organ mass (in mg) for the NI, I and IBz mice, at 14 dpi. (D) Frequency and absolute numbers of B (B220^+^) and T (CD3^+^) cells are shown for the NI, I and IBz mice, at 14 dpi. Data on parasite levels are shown as mean ± standard deviation, from one representative experiment (out of five) with n = 6 in I and n = 5 in IBz group. Splenocyte numbers and spleen cellularity are shown as data from two experiments, each with n = 3–4 in the NI group, n = 4–5 in the I group and n = 4 in IBz group. B and T cell numbers are depicted as data from one experiment, with n = 3 in the NI group, n = 4 in the I group and n = 5 in IBz group. Horizontal bars (B-D) represent the average for each group. ** P <0.01 unpaired Kruskal-Wallis (Dunn's post-test).
(TIF)

S4 Fig CD4 subset of benznidazole-treated mice bear increased frequency of effector CD44^+^CD62L^low^ cells. Frequency of CD44^+^CD62L^low^ within CD4^+^ subset was analyzed from the groups non-infected and non-treated (NI) with n = 4, infected non-treated (I) with n = 6 and infected and benznidazole-treated (IBz) with n = 5. Representative dotplots (CD62L *versus* CD44) are shown. Data are from one experiment analyzed on day 14 post-infection; the horizontal bars represent the average for each group, ** p <0.01, unpaired Kruskal-Wallis (Dunn's post-test).
(TIF)
