# Supplementary figures and images for: CD8low T cells expanded following acute Trypanosoma cruzi infection and benznidazole treatment are a relevant subset of IFN-γ producers
Source: PLoS Negl Trop Dis. 2020 Dec 21;14(12):e0008969. doi: 10.1371/journal.pntd.0008969 (PMC7785226; doi:10.1371/journal.pntd.0008969)

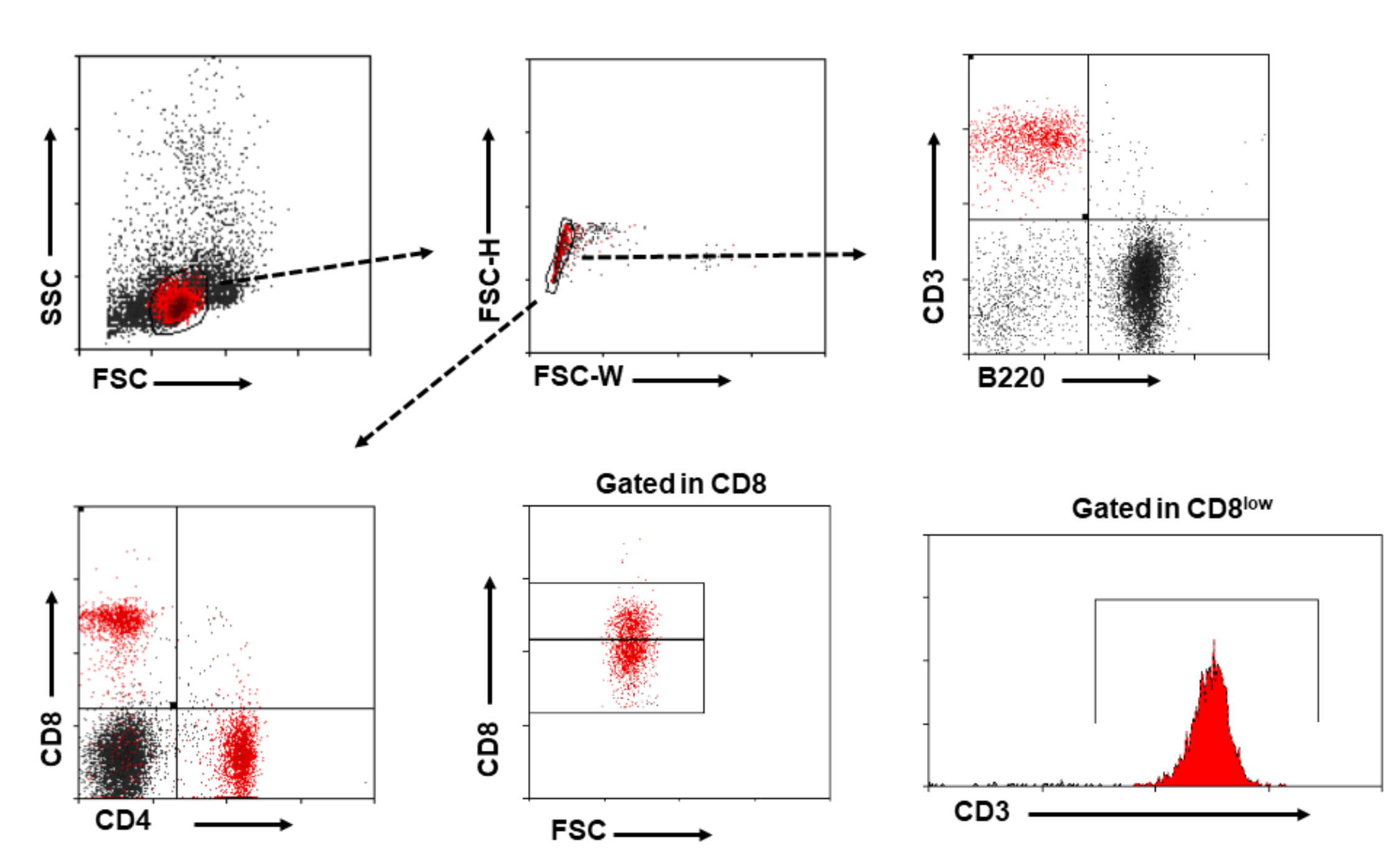

Supplement: S1 Fig — (TIF) [file pntd.0008969.s001.tif]

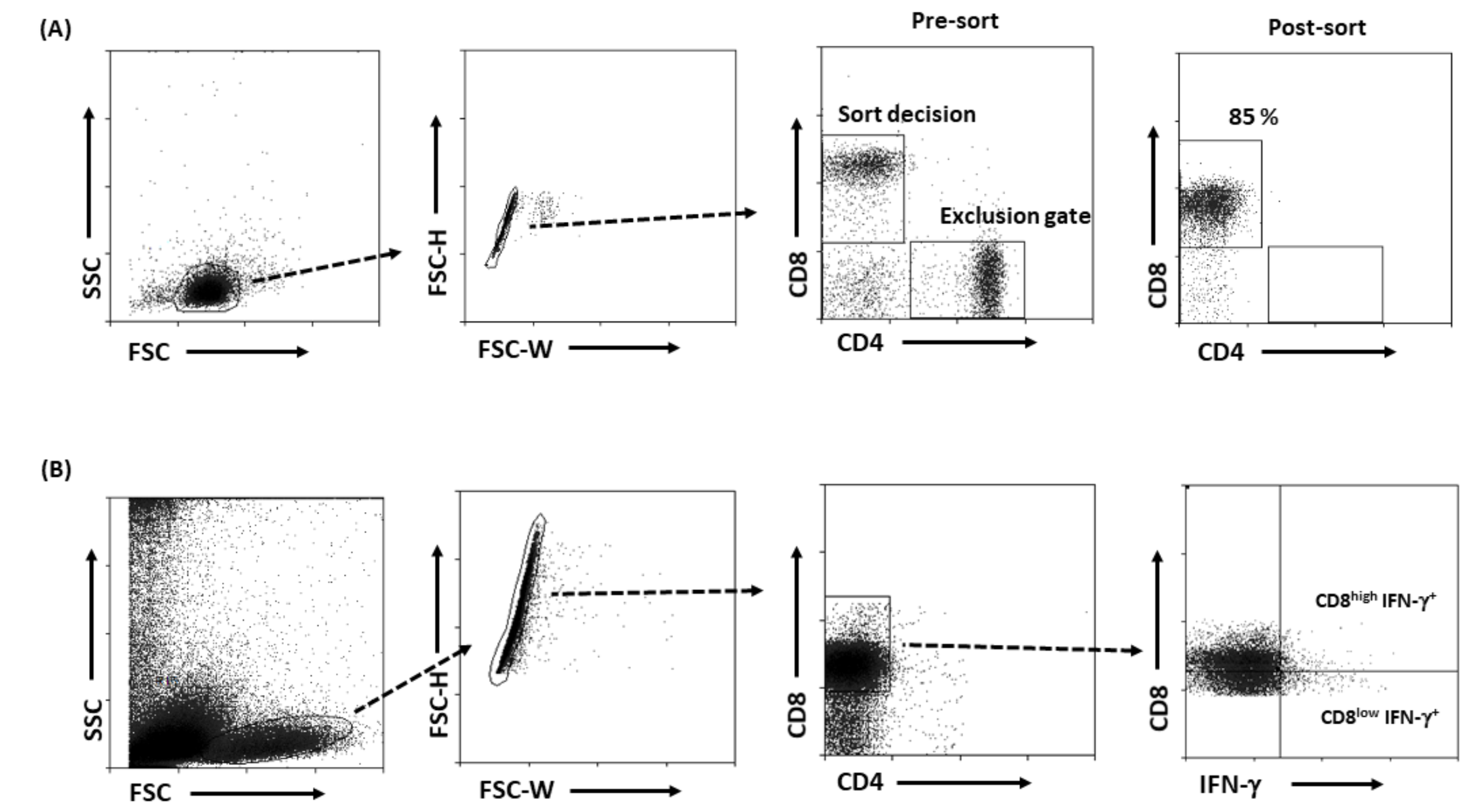

Supplement: S2 Fig — (TIF) [file pntd.0008969.s002.tif]

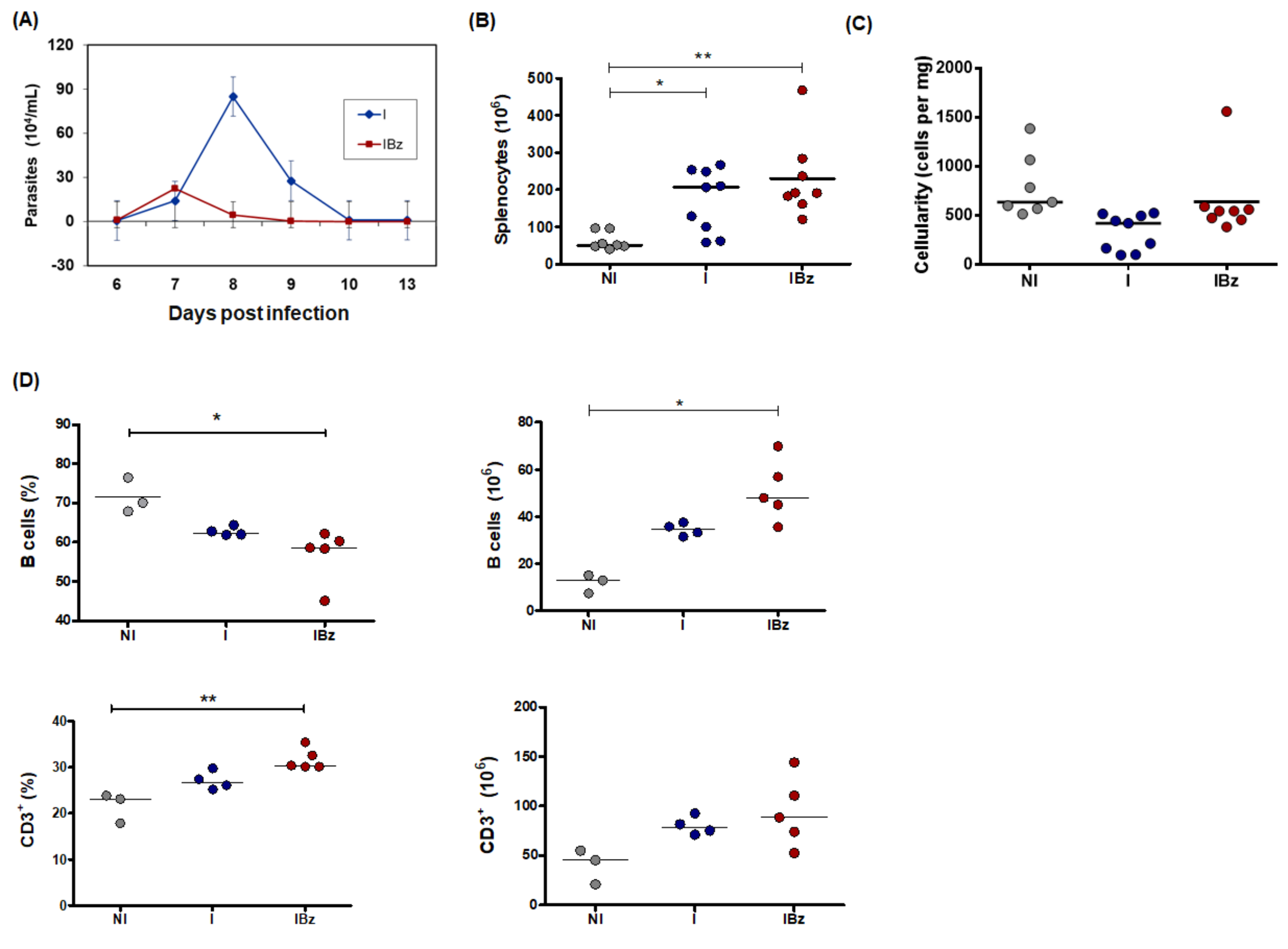

Supplement: S3 Fig — (A) Parasitemia levels were followed in mice from the infected non-treated (I) and infected and benznidazole-treated (IBz) experimental groups. (B) Absolute splenocyte number at 14 dpi was analyzed from groups of non-infected and non-treated (NI), I and IBz mice. (C) Spleen cellularity is shown as the ratio of splenocyte number (in millions) per organ mass (in mg) for the NI, I and IBz mice, at 14 dpi. (D) Frequency and absolute numbers of B (B220+) and T (CD3+) cells are shown for the NI, I and IBz mice, at 14 dpi. Data on parasite levels are shown as mean ± standard deviation, from one representative experiment (out of five) with n = 6 in I and n = 5 in IBz group. Splenocyte numbers and spleen cellularity are shown as data from two experiments, each with n = 3–4 in the NI group, n = 4–5 in the I group and n = 4 in IBz group. B and T cell numbers are depicted as data from one experiment, with n = 3 in the NI group, n = 4 in the I group and n = 5 in IBz group. Horizontal bars (B-D) represent the average for each group. ** P <0.01 unpaired Kruskal-Wallis (Dunn's post-test). (TIF) [file pntd.0008969.s003.tif]

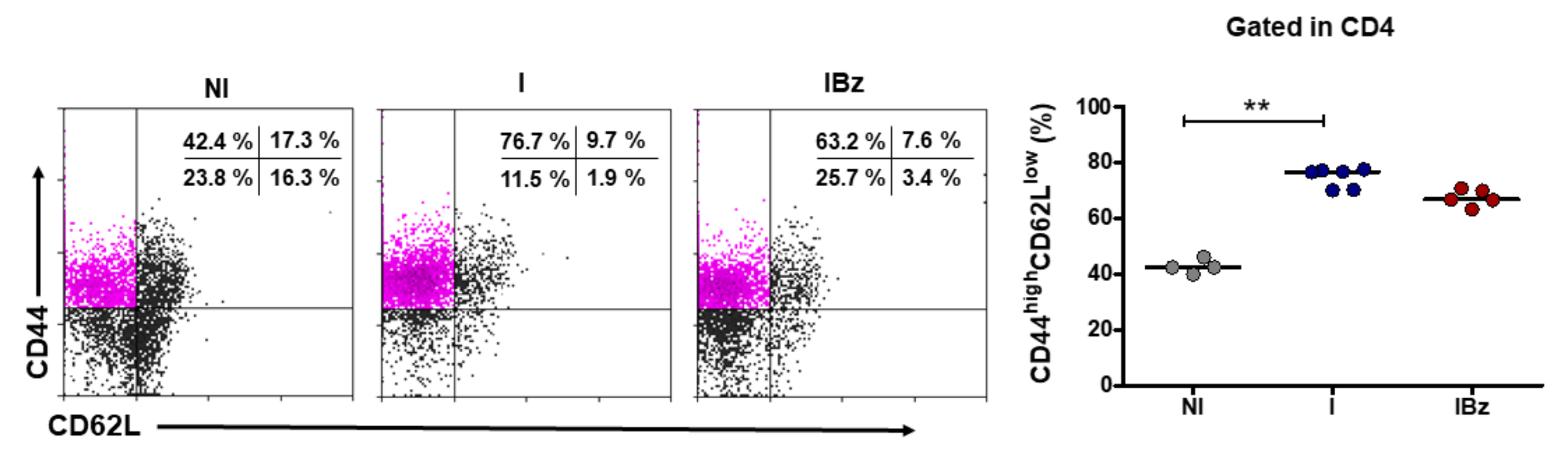

Supplement: S4 Fig — Frequency of CD44+CD62Llow within CD4+ subset was analyzed from the groups non-infected and non-treated (NI) with n = 4, infected non-treated (I) with n = 6 and infected and benznidazole-treated (IBz) with n = 5. Representative dotplots (CD62L versus CD44) are shown. Data are from one experiment analyzed on day 14 post-infection; the horizontal bars represent the average for each group, ** p <0.01, unpaired Kruskal-Wallis (Dunn's post-test). (TIF) [file pntd.0008969.s004.tif]
